# Comparison of two algorithms for determining beam weights and wedge filters

**DOI:** 10.1120/jacmp.v3i3.2562

**Published:** 2002-06-01

**Authors:** Jianrong Dai, Yunping Zhu

**Affiliations:** ^1^ Department of Radiation Oncology St. Jude Children's Research Hospital 332 N. Lauderdale Memphis Tennessee 38105‐2794; ^2^ Present address: Department of Radiation Oncology Cancer Institute (Hospital), Chinese Academy of Medical Sciences Beijing 100021 China

**Keywords:** beam weight, wedge filter, dose gradient, super‐omni wedge, treatment planning

## Abstract

This article compares two algorithms for determining beam weights and wedge filters for conformal treatment planning. One algorithm, which is based on dose‐gradient analysis, provides analytic formulas for determining beam weights, wedge angles, and collimator angles (i.e., wedge orientations) so that the dose distribution is homogeneous in the target volume. The second algorithm is based on the concept of the super‐omni wedge (i.e., the arrangement of two pairs of orthogonal nominal wedged beams), numerically optimize beam weights, wedge angles, and collimator angles so that the dose requirements to targets and organs at risk are satisfied to the best. Three clinical cases were tested. For the first case, both algorithms resulted in comparable homogeneous dose distributions in the target volume. For the second case, the second algorithm resulted in much lower doses to the eyes plus a better homogeneous dose distribution in the target volume. For the third case, only the second algorithm was applicable, and the treatment plan it developed met the prescribed requirements. The results show that the first algorithm is better in terms of feasibility, whereas the second is better in terms of applicability and the quality of treatment plans.

PACS number(s): 87.53.–j

## I. INTRODUCTION

One task of conformal treatment planning is to determine beam weights, wedge orientations, and wedge angles. This task can be accomplished manually through a trial‐and‐error procedure or automatically through an algorithm‐guided procedure. Manual adjustment of these parameters requires time and experienced planners. Even so, the resulting plans are at best feasible but not necessarily optimal, especially when multiple noncoplanar beams are included. Thus, developing algorithms for automatic determination of beam weights, wedge orientations, and wedge angles is desirable. Because dose distributions are linear functions of beam weights, automatic determination only of beam weights is straightforward and has been well investigated.^1^ ^–^ ^5^

However, dose distributions are neither linear functions of wedge angles nor linear functions of wedge orientations. Therefore, automatic determination not only of beam weights but also of wedge filters is much more difficult, and is still being investigated. Some methods (algorithms) are now available, which can be divided into two categories. One category is based on dose‐gradient analysis, which includes the method proposed by Sherouse^6^ and one algorithm proposed by the authors here.^7^ The authors' algorithm, which is based on Sherouse's method, provides analytic formulas for determining beam weights and wedge angles and wedge orientations for different kinds of beam arrangements. The second category of methods (algorithms) is based on mathematical optimization tools, which includes the algorithm proposed by Redpath *et al*.,^8^ the algorithm proposed by Oldham *et al*.,^9^ the algorithm proposed by a group of physicists in Stanford University,^10^ ^–^ ^12^ and one algorithm proposed by the authors here.^13^ The algorithm of Redpath *et al*. is the earliest among all those algorithms. It tries to exhaustively search all possible combinations of wedges with fixed angles of 15°, 30°, 45°, and 60°, and to select the combination with the lowest objective value as the optimal plan. The disadvantages of that algorithm are obvious: a limited number of wedge angles and fixed wedge orientations. The algorithm of Oldham *et al*.^9^ can optimize beam weights and wedge angles, and its application is illustrated with radiotherapy for cancer of prostate. The limitation of that algorithm is that wedge orientations need to be selected before optimization procedure. The algorithm of Stanford University is based on the concept of omni wedge. In the context of that algorithm, the problem of optimizing beam weights, wedge angles, and wedge orientations for *J* beams is transformed into the problem of optimizing beam weights for 3*J* nominal wedged beams. Because that kind of transformation needs to be done for each beam during each iteration of optimization procedure, it can be thought as a limitation of that algorithm. The authors' optimization algorithm is based on the concept of the super‐omni wedge. In the context of the algorithm, the problem of optimizing beam weights, wedge angles, and wedge orientations for *J* beams is transformed into the problem of optimizing beam weights for 4*J* nominal wedged beams. That kind of transformation only needs to be done once before optimization procedure.

Here we compare the performance of the authors' two algorithms in terms of feasibility, applicability, and the quality of treatment plans. The term “feasibility” means how an algorithm can be implemented; does it require integration with a treatment planning system. The term “applicability” means which kinds of clinical cases an algorithm can be applied to. The quality of treatment plans is evaluated with the dose uniformity in target volumes and the maximum doses received by organs at risk. Three clinical cases are tested. These cases are selected as the representatives of simple, somewhat complicated, and complicated cases. Because the two algorithms belong to two different categories of methods (algorithms) for automatic determination not only of beam weights but also of wedge filters, the performance of two categories of methods (algorithms) will be reflected by the comparison.

## II. METHODS AND MATERIALS

### A. Brief introduction to algorithm No. 1

#### 1. Definition of dose gradient

In the central part of an open beam, the dose gradient, $\overset{ˇ}{G}$, is pointed toward the source and lies parallel to the central axis of the beam.^6^ The magnitude of $\overset{ˇ}{G}$ is equal to the dose variation per unit depth. The effect of adding a wedge to the beam is to introduce a simple transaxial gradient ${\overset{ˇ}{G}}_{t}$, and the resulting dose gradient $\overset{ˇ}{G}$ is the vector sum of the inherent axial gradient vector ${\overset{ˇ}{G}}_{a}$ and the wedge‐induced transaxial gradient vector ${\overset{ˇ}{G}}_{t}$. The angle between $\overset{ˇ}{G}$ and ${\overset{ˇ}{G}}_{a}$ is the wedge angle $\theta^{w}$. Therefore, the relationship between the magnitudes of $\overset{ˇ}{G}$, ${\overset{ˇ}{G}}_{a}$, and ${\overset{ˇ}{G}}_{t}$ is given by

(1) $$G\sin\theta_{w}=G_{a}\tan\theta_{w}=G_{t}.$$

The necessary and sufficient condition for achieving a homogeneous dose over the target volume is met when the total vector sum of the dose gradients of the beams is zero everywhere in the target volume. That is to say, the following equation must be satisfied at any point in the target volume:

(2) $$W_{1}{\overset{ˇ}{G}}_{1}+W_{2}{\overset{ˇ}{G}}_{2}+L+W_{n}{\overset{ˇ}{G}}_{n}=0,$$

where $W_{i}$ is the relative contribution of the *i*th beam to the target dose, ${\overset{ˇ}{G}}_{i}$ is the dose gradient of the *i*th beam, and *n* is the number of beams.

When the number of beams and the direction of each beam are fixed, Eq. (2) can be satisfied by adjusting beam weights or by adjusting beam weights and adding wedges. Under ideal conditions, the dose distribution in the target volume will be homogeneous as long as Eq. (2) is satisfied at the intersection point of the central axes of the beams. However, clinical cases may differ from ideal conditions. Under such circumstances, beam parameters must still be adjusted manually.

Beam direction can be represented by a unit vector that is pointed toward the source along the central axis of a beam. When treatment machines are calibrated according to the coordinate systems defined by IEC 1217,^14^ the vector of beam direction in the treatment table system, ${\overset{ˇ}{B}}_{T}$, is given by

(3) $${\overset{ˇ}{B}}_{T}={\left(\sin\theta_{G}\cos\theta_{T},-\sin\theta_{G}\sin\theta_{T},\cos\theta_{G}\right)}^{T},$$

where $\theta_{G}$ is the gantry angle and $\theta_{T}$ is the table angle. It should be noted that the subscript *T* represents the coordinate system of treatment table while the superscript *T* is a matrix transpose operator.

### 2. Case of two angled beams

For a case of two angled beams, a plane *P* is set up to pass through the central axes of two beams, and a line, *AB*, in plane *P* is chosen to pass through the intersection point of the central axes of the beams (refer to Fig. 1 in Ref. 7). In the treatment table system, the hinge angle $\theta_{h}$ between the central axes of the two beams is determined by

**Figure 1:**
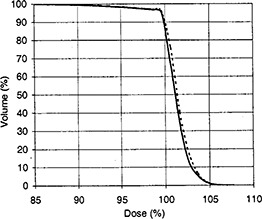
Dose‐volume histograms for the target for case 1. A solid curve indicates the result of algorithm No. 1, whereas a dashed curve indicates the result of algorithm No. 2.

(4) $$\theta_{h}=\cos^{-1}({\overset{ˇ}{B}}_{T1}\cdot {\overset{ˇ}{B}}_{T2}).$$

By adding wedges, we can shift the dose gradients of the two beams from their central axes to line *AB* in the opposite direction. Therefore, the wedge angle of each beam is equal to the angle between the central axis of this beam and line *AB*, and the following equation is tenable:

(5) $$\theta_{w1}+\theta_{w2}=180{}^{\circ}-\theta_{h}.$$

In clinical practice, we usually use a special case of Eq. (5), i.e.,

(6) $$\theta_{w1}=\theta_{w2}=90{}^{\circ}-\theta_{h}/2.$$

Using Eqs. (1) and (2), we can derive the formulas for determining beam weights [Eq. (7)] and collimator angles [Eq. (8)].

(7) $$W_{2}=W_{1}\cos\theta_{w2}/\cos\theta_{w1},$$

(8a) $$\begin{array}{c} \theta_{C1}=\cos^{-1}[\sin(\theta_{T2}-\theta_{T1})\sin\theta_{G2}/\sin\theta_{h}],\\ \text{or}\;\;\;\;\;360{}^{\circ}-\cos^{-1}[\sin(\theta_{T2}-\theta_{T1})\sin\theta_{G2}/\sin\theta_{h}], \end{array}$$

(8b) $$\begin{array}{c} \theta_{C2}=\cos^{-1}[\sin(\theta_{T1}-\theta_{T2})\sin\theta_{G1}/\sin\theta_{h}],\\ \text{or}\;\;\;\;\;360{}^{\circ}-\cos^{-1}[\sin(\theta_{T1}-\theta_{T2})\sin\theta_{G1}/\sin\theta_{h}], \end{array}$$

where $W_{1}$ and $W_{2}$ are beam weights and $\theta_{C1}$ and $\theta_{C1}$ are collimator angles. The collimator angles given by Eq. (8) represent the wedge orientations under the condition that the wedge orientations point towards the gantry (the wedge toes point towards the gantry) when the collimator angle is zero. The choice of $\theta_{C1}$ from the two values in Eq. (8a) and the choice of $\theta_{C2}$ from the two values in Eq. (8b) must assure that the two wedge heels are close to each other in the room's eye view (REV) of a treatment planning system.

#### 3. Case of three noncoplanar beams

For a case of three noncoplanar beams, a unit vector ${\overset{ˇ}{N}}_{T}$ is determined by

(9) $${\overset{ˇ}{N}}_{T}=\sum_{i=1}^{3}{\overset{ˇ}{B}}_{T_{i}}/\left|\sum_{i=1}^{3}{\overset{ˇ}{B}}_{T_{i}}\right|$$

and a plane *P* is set up to pass through the intersection point of all of the central axes of the beams with ${\overset{ˇ}{N}}_{T}$ as its normal direction (refer to Fig. 3 in Ref. 7). The angle between the central axis of the *i*th beam and the normal direction of the plane $P,\beta_{i}$, is given by

**Figure 3:**
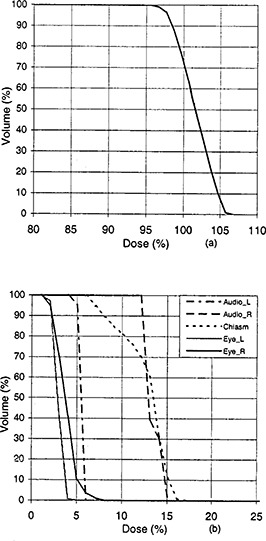
Dose‐volume histograms for the planning target volume (a) and the organs at risk (b) for case 3.

(10) $$\beta_{i}=\cos^{-1}({\overset{ˇ}{B}}_{T_{i}}\cdot {\overset{ˇ}{N}}_{T}).$$

The dose gradient of each beam can be shifted from the central axis to the plane *P* by adding a wedge of angle $\beta_{i}$ so that the dose gradients of the three beams are distributed in an angle larger than 180° in the plane *P*. Then, the formula for beam weights is

(11) $$W_{2}=W_{1}\frac{\sin\alpha_{13}\sin\beta_{2}}{\sin\alpha_{32}\sin\beta_{1}},\;\;\;\;\;\;\;W_{3}=W_{1}\frac{\sin\alpha_{21}\sin\beta_{3}}{\sin\alpha_{32}\sin\beta_{1}},$$

where $\alpha_{ij}$ is the angle between the dose gradients of the *i*th and the *j*th beams.

The wedge angle of the *i*th beam is given by

(12) $$\theta_{wi}=90{}^{\circ}-\beta_{i}.$$

The collimator angle of the *i*th beam is given by

(13) $$\theta_{C_{i}}=180{}^{\circ}-\cos^{-1}({\overset{ˇ}{I}}_{Twi}\cdot {\overset{ˇ}{N}}_{T}/\sin\beta_{i})\;\;\;\;\;\;\text{or}\;\;\;\;\;180{}^{\circ}+\cos^{-1}({\overset{ˇ}{I}}_{Twi}\cdot {\overset{ˇ}{N}}_{T}/\sin\beta_{i}),$$

where ${\overset{ˇ}{I}}_{Twi}$ represents the wedge orientation when the collimator angle is zero and is given by ${\overset{ˇ}{I}}_{Twi}={\left(\sin\theta_{Ti},\cos\theta_{Ti},0\right)}^{T}$.

The choice of $\theta_{C1}$ from the two values of Eq. (13) is determined so that the angle between the wedge orientation and the normal direction of plane *P* is greater than 90°.

### B. Brief introduction to algorithm no. 2

#### 1. The concept of the super‐omni wedge

The super‐omni wedge is defined as the arrangement of two orthogonal pairs of nominal wedged beams. In each pair of nominal wedged beams, the wedge orientations are opposite each other. If the weights of four beams are equal, the combined dose distribution will be as flat as that of an open beam. Otherwise, the combined dose distribution will be wedged. When beam weights are adjusted, the effective wedge angle can vary from zero to the maximum effective wedge angle, and the effective wedge orientation can vary from 0° to 360°. The super‐omni wedge is an extension of the omni wedge: the arrangement of one open beam and two orthogonal nominal wedged beams.1516 The beams for the super‐omni wedge can be transformed into the beams for the omni wedge. This transformation is used to determine the effective wedge orientation and wedge angle for the super‐omni wedge.

#### 2. Optimization of beam parameters

By using the concept of the super‐omni wedge, we can transform the problem of optimizing beam weights, wedge orientations, and wedge angles for *J* beams into the problem of optimizing beam weights for 4*J* nominal wedged beams. When the relative dose distribution is calculated for every nominal wedged beam with unity weight at a common reference point *a priori*, the absolute dose distribution for all beams can be calculated by weighted summation of the relative dose distributions. Therefore, there is no need to recalculate the relative dose distributions during the optimization process.

The optimization goal is to minimize the dose inhomogeneity in the target volume under the constraints that the doses received by the organs at risk must not exceed prescribed upper limits. The optimization problem is solved with a successive quadratic‐programming algorithm.

After the weights of the nominal wedged beams have been determined, each group of four nominal wedged beams is transformed into a wedged beam by using the virtual wedge function of our accelerators. Therefore, the results for Algorithm No. 2 in the next section are obtained with the transformed beams instead of the original nominal wedged beams.

## III. RESULTS

To compare the two algorithms, we selected three clinical cases: a case of two angled beams, a case of three noncoplanar beams, and a case of seven noncoplanar beams. The three cases were typical in clinical practice, and selected from over 30 patients recently treated in our department. They represented simple, somewhat complicated, and complicated cases. Both algorithms were applied to the first two cases. However, only algorithm No. 2 was applied to the third case, because algorithm No. 1 does not provide formulas for a case that has more than three beams. When algorithm No. 1 was used, the beam weights, wedge angles, and collimator angles were manually calculated with a calculator. In contrast, when algorithm No. 2 was used, the beam weights, wedge angles, and collimator angles were determined by our in‐house optimization program. For all three cases, beam energy was 6 MV (Primus, Siemens Medical Systems, Concord, CA). When the collimator angle was zero, the wedge orientation was set towards the gantry. Treatment plans were designed on a 3D treatment planning system (PLUNC, Radiation Oncology Department, University of North Carolina, Chapel Hill, NC).

### A. Case 1: Two angled beams

A plan of two angled beams was designed for the Phase I treatment of a patient with medullo‐blastoma close to the left ear. The patient was placed into the prone position. One beam came from the right‐posterior‐inferior direction with a gantry angle of 290° and a table angle of 335°. Another came from the left‐posterior‐inferior direction with a gantry angle of 70° and a table angle of 20°. With algorithm No. 1, the vectors of beam directions as determined by Eq. (3) were (–0.852, –0.397, 0.342) and (0.883, –0.321, 0.342), and the hinge angle between the two beams was 120°, as determined by Eq. (4). The wedge angle of each beam, as determined by Eq. (6), should be 30°. However, we chose to use a wedge angle of 40° for both beams; the increased wedge angle served as a missing tissue compensator. The beam weights were equal. As determined by Eq. (8), the collimator angles of the first beam were either 40° or 320°, as were those of the second beam. By observing the REV of the PLUNC system, we judged that the collimator angle of the first beam should be 40°, whereas that of the second should be 320°. With algorithm No. 2, the optimization goal was set as a uniform dose distribution in the target volume. The optimized beam parameters were as follows: beam weights 0.417 and 0.583; wedge angles 54° and 30°; collimator angles 19° and 291°. There were some differences between the beam parameters predicted by the two algorithms. However, the dose volume histograms for the target volume showed that the two algorithms resulted in comparable dose distributions (Fig. 1).

### B. Case 2: Three noncoplanar beams

**Figure 2:**
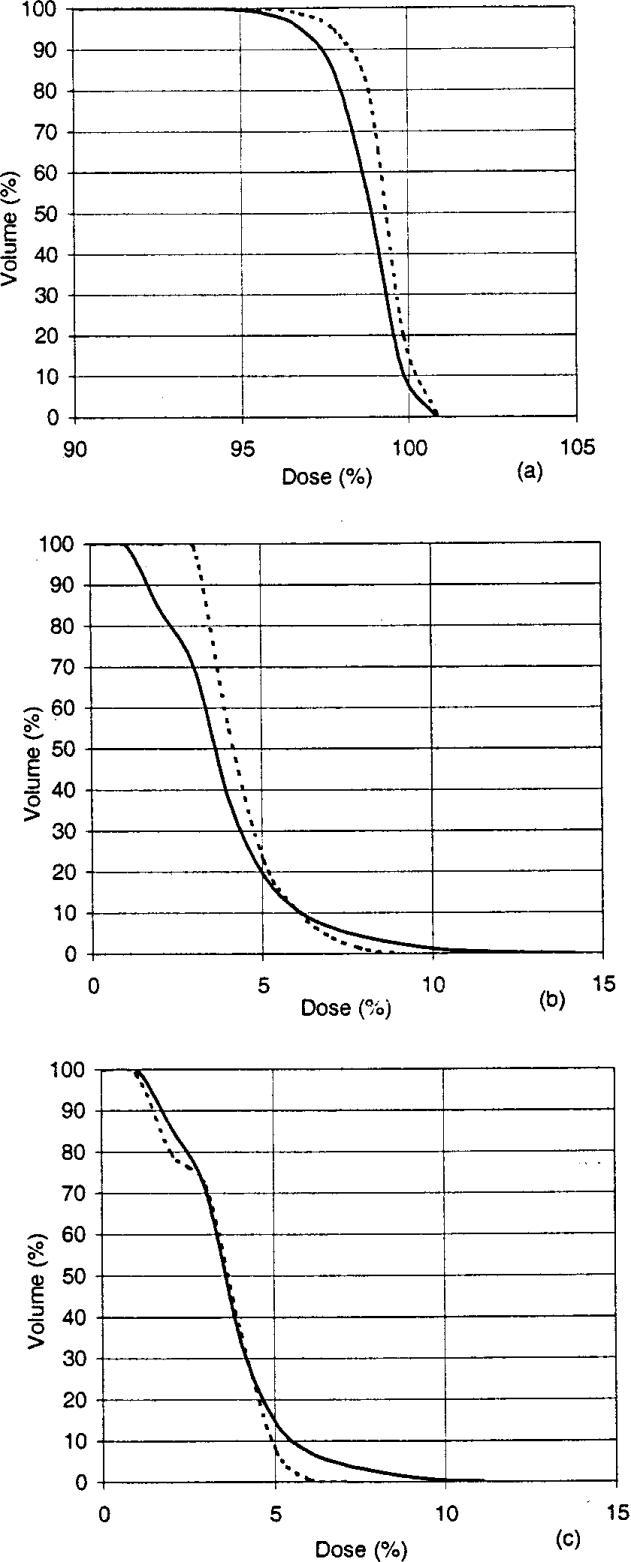
Dose‐volume histograms for the target (a), the left eye (b), and the right eye (c) for case 2. Solid curves indicate the results of algorithm No. 1, whereas dashed curves indicate the results of algorithm No. 2.

Three noncoplanar beams were manually arranged to treat a patient with a tumor of the hypothalamus. The patient was placed into the supine position. The isocenter of all three beams was placed at the center of the target, and the beams were distributed symmetrically around the inferior‐superior axis. The gantry angles were $\theta_{G1}=111{}^{\circ},\theta_{G2}=249{}^{\circ}$, and $\theta_{G3}=315{}^{\circ}$, and the couch angles were $\theta_{T1}=229{}^{\circ},\theta_{T2}=131{}^{\circ}$, and $\theta_{T3}=90{}^{\circ}$. When algorithm No. 1 was used, the inferior‐superior axis represented the normal direction ${\overset{ˇ}{N}}_{T}$ as determined by Eq. (9). The plane $X_{T}-Z_{T}$ was the plane *P*. The angle between the central axis of each beam and the normal direction was 45°, i.e., $\beta_{1}=\beta_{2}=\beta_{3}=45{}^{\circ}$. The angle between the dose gradients of every two beams was 120°, i.e., $\alpha_{ij}=120{}^{\circ}(i=1,2,3;j=1,2,3;i\#j)$. The weights of all beams were determined to be equal by Eq. (11), and the wedge angle of each beam was 45° as derived by Eq. (12). In this example, the incidence of each of the three beams was nearly perpendicular to the patient's skin surface. Therefore, the calculated wedge angle for each beam did not need to be changed. The collimator angles of the three beams were 338°, 22°, and 270° as derived by Eq. (13) and by observing the REV When algorithm No. 2 was used, the optimization goal was set as follows: target dose equal to 54 Gy, and dose to the eyes less than 6 Gy. The optimized beam parameters were as follows: beam weights 0.280, 0.456, and 0.264; wedge angles 58°, 46°, and 24°; collimator angles 359°, 12°, and 21°. The dose‐volume histograms showed that algorithm No. 2 resulted in a slightly more homogeneous dose distribution in the target volume and a much lower maximum dose to the eyes (Fig. 2). The target dose ranged from 50.7 Gy (93.8%) to 54.4 Gy (100.8%) for algorithm No. 1 and from 51.7 Gy (95.8%) to 54.5 Gy (100.9%) for algorithm No. 2. The maximum dose to the left eye was 7.6 Gy (14.1%) for algorithm No. 1 and 4.9 Gy (9.1%) for algorithm No. 2. The maximum dose to the right eye was 6.0 Gy (11.1%) for algorithm No. 1 and 3.3 Gy (6.1%) for algorithm No. 2.

### C. Case 3: Six noncoplanar beams

Six noncoplanar beams were arranged to treat a patient with a malignant neoplasm in the right parietal region of the brain. The patient was placed into the supine position. The apertures of all beams were conformal to the planning target volume when a multileaf collimator with a 0.8 cm margin was used under the condition that the eyes were blocked from primary radiation with a 0.3 cm margin of avoidance. Because algorithm No. 1 has no formulas for a case that requires more than three beams, only algorithm No. 2 was used for this patient. The planning criteria specified a prescribed dose of 50.4 Gy to the planning target volume and an upper dose limit of 5 Gy to the eyes, 5 Gy to the left auditory, 8 Gy to the right auditory, and 10 Gy to the chiasm. Table I lists beam setup data; the gantry angles and table angles were manually selected, whereas the collimator angles, wedge angles, and beam weights were determined through optimization. Fig. 3 shows the dose‐volume histograms for the planning target and the organs at risk. The dose in the planning target volume ranged from 48.2 Gy (95.6%) to 53.8 Gy (106.7%). The maximum doses were 2.5 Gy (5.0%) to the left eye, 4.1 Gy (8.1%) to the right eye, 3.0 Gy (6.0%) to the left auditory, 7.6 Gy (15.1%) to the right auditory, and 8.6 Gy (17.1%) to the chiasm; these doses were below the prescribed dose limits.

**Table I:** Beam setup data for the case of six noncoplanar beams. $\theta_{g}$, gantry angle; $\theta_{t}$, table angle; $\theta_{c}$, collimator angle; $\theta_{w}$, wedge angle; W, weight. All angles are expressed in degrees.

No.	1	2	3	4	5	6
$\theta_{g}$	352	23	83	316	286	252
$\theta_{t}$	311	282	289	347	246	344
$\theta_{c}$	0	180	180	0	178	118
$\theta_{w}$%	48	60	60	60	59	52
W	0.091	0.254	0.081	0.070	0.175	0.329

## IV. DISCUSSION AND CONCLUSION

The performance of algorithms can be evaluated in terms of feasibility, applicability, and the quality of treatment plans. As mentioned in the Introduction section, the term “feasibility” means how an algorithm can be implemented; does it require integration with a treatment planning system. The term “applicability” means which kinds of clinical cases an algorithm can be applied to. The quality of treatment plans is evaluated with the dose uniformity in target volumes and the maximum doses received by organs at risk. Algorithm No. 1 provides analytic formulas. We can determine beam weights and wedge filters with a calculator. However, we cannot implement algorithm No. 2 manually; we must use complicated mathematical tools, sophisticated computer programming, and must eventually integrate the algorithm into a treatment planning system. If a commercial treatment planning system is used, it is impossible for the user to integrate the algorithm. Therefore, algorithm No. 1 is more feasible than algorithm No. 2 mainly in that it does not require integration with a planning system. However, algorithm No. 1 provides analytic formulas only for cases requiring two or three beams. For cases requiring more than three beams, the algorithm provides only suggestions, one of which is to divide beams into groups of two or three beams. Moreover, it cannot directly deal with dose constraints to organs at risk or with the effect of irregular surface and inhomogeneity on dose gradient. Algorithm No. 2, on the other hand, has no such limitations. Therefore, algorithm No. 2 is more applicable than algorithm No. 1 mainly because algorithm No. 2 can be applied to a broader class of clinical cases.

The quality of treatment plans is the most important issue. The two algorithms produce comparable treatment plans for simple cases like the first case described above. Algorithm No. 2 produces better treatment plans for somewhat complicated cases like the second case described above. Algorithm No. 2 can produce treatment plans for complicated cases like the third case described above, whereas it is difficult for algorithm No. 1 to deal with such cases. For some clinical cases, it is necessary to adjust the beam parameters predicted by algorithm No. 1, as we did for the first case described above. This kind of adjustment is not required by algorithm No. 2. Therefore, the quality of the treatment plans produced by algorithm No. 2 is generally better than that of plans produced by algorithm No. 1.

In conclusion, this study compared two algorithms for determining beam weights and wedge filters for conformal treatment planning. One algorithm, based on dose‐gradient analysis, provides analytic formulas for determining beam weights and wedge filters. The second, based on the concept of the super‐omni wedge, numerically optimizes beam weights and wedge filters. Between them, the first algorithm is better in terms of feasibility, whereas the second is better in terms of applicability and the quality of treamtnet plans.

## ACKNOWLEDGMENTS

This investigation was supported by Grant No. R29 CA65606, Cancer Center Support CORE Grant No. P30 CA 21765 from the National Cancer Institute, and by the American Lebanese Syrian Associated Charities (ALSAC).
